# Development and initial validation of the psychological capital scale for nurses in Chinese local context

**DOI:** 10.1186/s12912-022-01148-x

**Published:** 2023-02-02

**Authors:** Xiangyu Lu, Lina Wang, Guifang Xu, Haixia Teng, Jing Li, Yufang Guo

**Affiliations:** 1 Hebei Provincial Mental Health Center, Baoding, Hebei People’s Republic of China; 2 Hebei Key Laboratory of Major Mental and Behavioral Disorders, Baoding, Hebei People’s Republic of China; 3 The Sixth Clinical Medical College of Hebei University, Baoding, Hebei People’s Republic of China; 4 Zibo Municipal Hospital, Zibo, Shandong People’s Republic of China; 5 The Fifth People’s Hospital of Jinan, Jinan, Shandong People’s Republic of China; 6 School of Nursing and Rehabilitation, Shandong University, Jinan, Shandong People’s Republic of China

**Keywords:** Chinese local context, Nurses, Psychological capital, Scale

## Abstract

**Background:**

Psychological capital is affected by different cultures and professional characteristics and its constituent dimensions and evaluation tools are heterogeneous. There is a lack of measurements for assessing nurses’ psychological capital considering nursing professional characteristics and Chinese cultural impacts.

**Aims:**

To develop a psychological capital scale that conforms to the Chinese cultural background and the characteristics of nursing profession, and evaluate the preliminary validation of the Nurses Psychological Capital Scale.

**Methods:**

Nurses were conveniently recruited from two tertiary hospitals, Hebei, China. The research process included three steps: item development (Delphi survey and pilot survey), scale development (item analysis and exploratory factor analysis), scale validation (reliability and validity test).

**Results:**

Exploratory factor analysis and confirmatory factor analysis showed that the 43-item scale comprised three factors (work task-oriented psychological capital, interpersonal relationship-oriented psychological capital and learning development-oriented psychological capital). Exploratory factor analysis showed the factor loadings ranging from 0.460 to 1.029. Three factors explained 68.71% of the variance. Confirmatory factor analysis showed an adequate model fit (*x*^2^/*df* =2.839, RMR = 0.041, RMSEA = 0.078, IFI = 0.872, TLI = 0.863, CFI = 0.871, PNFI = 0.768). The Cronbach’s α for the scale was 0.975. The item-level content validity index (I-CVI) was 0.83 ~ 1.00, scale-level average content validity index (S-CVI/Ave) was 0.988.

**Conclusion:**

The Nurse Psychological Capital Scale had good reliability and validity, which is a reliable evaluation measure for assessing psychological capital among nurses.

**Supplementary Information:**

The online version contains supplementary material available at 10.1186/s12912-022-01148-x.

## Background

Psychological capital (PsyCap) was firstly proposed by Goldsmith in the 1990s [1]. After years of exploration by researchers, it has become a hot spot in the fields of organizational behavior and human resource management at home and abroad, and has attracted interests of researchers from psychology, management, medicine, education, sociology and other fields. As an important psychological resource, PsyCap reflects a positive psychological state that individuals show in the process of growth and development, which is composed of four core components: self-efficacy (the confidence to make necessary efforts to achieve success in the face of challenging work conditions); optimism (positive attribution to present and future success); hope (persevere in the goal and adjust the way to achieve the goal when necessary to achieve success); resilience (the ability to persevere in the face of adversity and problems, to recover quickly and to succeed) [2]. Empirical evidences indicate that PsyCap could improve employees’ job adaptability, initiative, proficiency and overall job performance [3]. In addition, PsyCap has negative correlations with employees’ attitudes such as cynicism, turnover intention, work stress and anxiety. It is negatively correlated with deviant behavior [4].

Based on the standards of positive organizational behavior (POB), Luthans et al. [5] incorporated hope, resilience, optimism and self-efficacy into the PsyCap structure and developed a PsyCap questionnaire (PCQ-24). As a universal questionnaire, PCQ-24 shows good reliability and validity and is widely used by scholars. The Chinese version of PCQ-24 had been widely used in the communication enterprise employees [6, 7], teachers [8], nurses [9], which showed good reliability with Cronbach’s α were over 0.8. However, PCQ-24 has been developed based on employees from the western organizational context, which may be not suitable for employees from other cultural background. Luthans et al. [10] reported that different cultures influenced individuals’ access to resources, therefore, psychological abilities were arose differently in individualistic and collectivist cultures. Western culture has been described as traditionally individualistic. It regards the individual as an independent and autonomous entity, pays attention to individual goals, and does not overemphasize collectivism. Conversely, China is a typical collectivist cultural country [11]. It pays more attention to collective goals and group values. The Chinese are more able to make sacrifices and contributions for the collective and put collective interests above individual interests [11]. As a collection of positive core psychological elements, the concept of psychological capital originates from the United States. The United States is a country with typical individualism orientation, so its dimension composition may be different in China.

As the important effects of PsyCap on individuals’ work attitude, behavior and performance, more and more researchers have explored the structure of PsyCap in different groups based on different cultural backgrounds and professional characteristics. Several kinds of special evaluation measurements have been developed to evaluate PsyCap. Ke et al. [12] developed the PsyCap structure for employees based on the Chinese cultural background, which included work task-oriented psychological capital (self-efficacy and courage, optimism and hope, enterprising and tenacious) and guanxi-oriented psychological capital (modesty and stability, tolerance and forgiveness, courtesy and Thanksgiving dedication). Rego et al. [13] found that the structure of PsyCap for Portuguese civil servants included self-efficacy, optimism, resilience, waypower and willpower. Mao et al. [14] explored the PsyCap structure for primary and secondary school teachers, and found that task-oriented psychological capital (self-efficacy, enterprising spirit, hope, optimism and resilience) and interpersonal emotional-oriented psychological capital (enthusiasm, humor, love and gratitude, fairness and integrity) together constitute the core structure of PsyCap. Zhang et al. [15] followed the four factor structure of PsyCap and compiled a questionnaire to surveyed college students’ PsyCap. Lou et al. [16] construct the PsyCap Scale for male nurses in Taiwan with 16 items and four factors based on the relevant literature: hope, optimism, resiliency, and self-efficacy. Cui et al. [17] developed the PsyCap questionnaire for cancer patients, which also included four structures: self-efficacy, hope, resilience and optimism. Luo et al. [18] revised PCQ-24 in combination with the actual situation of nursing work in China, and finally retained 20 items. At present, it has been widely verified by nurses in China. However, the questionnaire still adopts the four factor composition of PsyCap proposed by Luthans, without considering the different impacts of Chinese and Western culture on the structure of psychological capital.

Western culture pursues self-worth, while Chinese traditional culture is a kind of moral culture that emphasizes the humanistic spirit, the golden mean and harmony [19]. Taoism in China pursues the harmony and unity between man and nature, pays attention to life care, and believes that everything in the world should be natural and respect the form and value orientation of individual survival, and put human life in the first place [20]. This concept of humanistic care is highly consistent with the work core of nursing, which is silent dedication, respect for patients, and selfless care for life [21]. Confucianism advocates an active attitude, and its optimistic, aggressive and indomitable spirit also imperceptibly affects the psychological shaping of nurse groups [22]. In addition, western culture emphasizes individual independence, while China emphasizes interdependent culture, which focuses on adjusting the relationship between individuals and others to maintain the harmony of social relation [12]. Therefore, the establishment and maintenance of interpersonal relations between Chinese employees may be different from those in western countries. Moreover, considering the professionalism of nursing, nurses not only complete clinical care with high quality, but also need good communication skills to form good relationships with doctors, patients and other staffs. Therefore, nurses need to have good interpersonal skills. In conclusion, influenced by different cultures, the positive psychological composition of nurses in China includes not only the PsyCap structure of Western nurses (self-efficacy, hope, resilience, optimism), but also the characteristics of sense of community, dedication, respect, inclusiveness and so on, which is different from original PsyCap structure.

Due to the influence of Chinese cultural characteristics on nurses’ PsyCap, the existing scales can not well evaluate nurses’ PsyCap. Therefore, this study aimed to develop a PsyCap scale for Chinese nurses through literature review and Delphi method, and evaluate the reliability and validity of the new PsyCap scale.

## Methods

With the support and help of the collected hospital administrators, the researchers have obtained the informed consents from the subjects. From May to December 2021, the Nurse Psychological Capital Scale (NPCS) was developed and verified in a three-stage method, including item development, scale development and scale validation. In the item development stage, we initially formed the item pool of the scale through literature analysis, semi-structured interview and Delphi survey. After content validity was tested by experts, a pilot survey of the items was conducted with a sample of nurses. In the scale development stage, a main survey was conducted by applying the NPCS scale to nurses. Item screening through item analysis and exploratory factor analysis (EFA). Finally, the reliability and validity of the scale were tested.

### Phase 1 item development

#### Item generation

Based on the theory of POB and the concept of PsyCap, the research group collected data through literature analysis and semi-structured interview. Using the method of thematic analysis, a second-order three factors structural framework of nurses’ psychological capital was initially constructed. Nurses’ PsyCap is defined as a kind of exploitable and measurable positive psychological power that can promote individual competitiveness in the process of completing nursing work, communicating effectively and planning personal development under the Chinese cultural background [23]. It includes work task-oriented psychological capital (calmness, work immersion, resilience, optimism, self-efficacy, professional responsibility, adaptation), interpersonal relationship-oriented psychological capital (dedication, inclusiveness, modesty, sense of community), learning development-oriented psychological capital (reflection, learning to use, initiative, innovation) [23].

In this study, the structure of nurses’ PsyCap was taken as the basis for the dimension setting of the NPCS. Work task-oriented psychological capital, interpersonal relationship-oriented psychological capital and learning development-oriented psychological capital were taken as the first-level dimensions of the NPCS. Calmness, work immersion, resilience, optimism, self-efficacy, professional responsibility, adaptation, dedication, inclusiveness, modesty, sense of community, reflection, learning to use, initiative, innovation were taken as the second-level dimensions of the NPCS. The typical declarative sentences collected by the research group in the early stage were used as the main source of the third-level items of the scale. After repeated discussion by the research group, the NPCS was initially formed, which included 3 first-level dimensions, 15 second-level dimensions and 70 third-level items. A two-round Delphi survey was used to obtain the expert reviews of the initial items. Seventeen experts in nursing psychology, nursing education, nursing management and clinical nursing were consulted. Among them, 14 experts have worked for more than 10 years and 14 have senior professional titles. Through group discussion and two rounds of Delphi, 2 second-level dimensions of “adaptation” and “initiative” and 17 items were deleted. A scale consisting of 3 first-level dimensions (work task-oriented psychological capital, interpersonal relationship-oriented psychological capital, learning development-oriented psychological capital), 13 second-level dimensions (calmness, work immersion, resilience, optimism, self-efficacy, professional responsibility, dedication, inclusiveness, humility, sense of community, critical reflection, initiative, innovation) and 53 third-level items was finally formed.

#### Content validity test

In this study, six experts engaged in nursing psychology, nursing education and nursing management were invited to assess the content validity. Experts were required to rate correlations between the content described in each item and the measurement dimensions and concepts (1–4 points for “not relevant” to “very relevant”), and gave modification comments on the items. Item level content validity index (I-CVI) and scale level average content validity index (S-CVI/Ave) were used to evaluate content validity. S-CVI/Ave > 0.90, and when the number of experts≤5, I-CVI = 1.00, and when the number of experts> 5, I-CVI ≥ 0.78 [24].

#### Pilot survey

A small sample pre survey was conducted among 20 nurses from a tertiary hospital in Hebei, China, to check whether the scale has any content that was difficult to understand, repeated or unclear.

### Phase 2 scale development

Nurse participants were recruited from two third-A-grade general hospitals in Hebei Province, China. Convenience sampling method was used. Registered nurses who had obtained the national nurse qualification examination certificate and worked for 1 year or more were included in the study. Considering that the sample size of factor analysis is 5 ~ 10 times the number of items, 619 nurses were recruited in this study [25]. Among them, 30 nurses completed the same questionnaire within 2 weeks. The final sample was randomly divided into two parts by SPSS 22.0 software (select cases - precise random sampling). Three hundred and nineteen samples were used for scale development, 300 samples were used for scale validation.

#### Item analysis

Four methods were used to test the appropriateness or reliability of the scale or individual items: critical ratio (CR), item total correlation coefficient, Cronbach’s α coefficient method, commonality and factor load test method, the results can be used as the basis for item selection or modification. CR > 3, *P*-value of high-low-27% group comparison less than 0.05, and item-total correlation coefficient more than 0.40, the Cronbach’s α of the whole questionnaire did not increase after deleting an item, commonality> 0.2, factor load> 0.45 were retained, and the others were eliminated [25].

#### Exploratory factor analysis (EFA)

EFA was performed to identify the factor structure of the NPCS via SPSS 22.0. In order to ensure the validity of factor analysis of NPCS, we conducted the Kaiser-Meyer-Olkin (KMO) test and Bartlett’s test of sphericity. A KMO coefficient greater than 0.9 indicates a very good fit; 0.8 ~ 0.9 indicates a good fit; 0.7 ~ 0.8 indicates a general fit; 0.6 ~ 0.7 indicates a barely fit; and less than 0.6 indicates a poor fit in factor analysis. Bartlett’s test of sphericity (*p* < 0.05) is suitable for factor analysis. The principal component analysis method is used to extract the factors. The choice of the rotation axis mode is determined according to the correlation coefficient between the factors. If the correlation coefficient between the factors is greater than 0.3, the oblique rotation method is used for rotation, otherwise the orthogonal rotation axis is used. Eigenvalue> 1.0 and the cumulative variance contribution rate should reach 50% ~ 60% were considered as the criteria. The criteria for deleting items are: item factor load is less than 0.4; the load of the item on two or more factors is larger than 0.4; the load difference between the two factors is less than 0.25; a single factor contains less than 3 items [26, 27].

### Phase 3 scale validation

#### Confirmatory factor analysis (CFA)

CFA was performed to validate the factor structure of the NPCS developed in exploratory factor analysis via AMOS 24.0. Generally, the indicators used to judge the fitting degree of the model are the preliminary fit criteria of the model: the error variance of the model parameters is a positive number; the t-test of the estimated parameters was statistically significant (*p* < 0.05); the standard errors of parameter estimates are very small; factor load is between 0.50 and 0.95. Overall model fit: Chi- square degree of freedom ratio (*x*^2^/*df*), Generally, it is considered that *x*^2^/*df* < 3, indicating that the fitting of the model is very good. When 3< *x*^2^/*df* < 5, indicating that the model is acceptable; The Root Mean Square Error of Approximation (RMSEA) < 0.05 indicates that the model fits well, and the fitting degree of 0.05 < RMSEA< 0.08 is good; Root Mean Square Residual (RMR) < 0.05 indicates that the model fits well; The Incremental Fit Index (IFI), Tucker Lewis Index (TLI) and Comparative Fit Index (CFI) above 0.9 are good fit, and above 0.8 are reasonable fit; The parsimony-adjusted normed fit index (PNFI) > 0.5 indicates that the model is acceptable [28, 29].

#### Convergent and discriminant validity

The combination reliability (CR) and average variance extracted (AVE) were taken as the indicators to evaluate the convergence validity [30]. Generally, it is considered that CR > 0.7, AVE > 0.5, and the convergence validity is good. When 0.36 < AVE < 0.5, the convergence validity is acceptable [31, 32]; The discriminant validity is compared with the square root of AVE using the correlation coefficient between factors. If the correlation coefficient between factors is less than the square root of the corresponding AVE, it indicates that the scale has a good discriminant validity [30].

#### Criterion-related validity

Criterion-related validity refers to the relationship between a research tool and other measurement standards. The higher the correlation coefficient, the better the validity of the measurement tool [33]. Work engagement Scale was selected for criterion related validity test. According to the job demand-resource model, there was a positive correlation between personal resources and work engagement. Increased personal resources can promote work engagement. Empirical research shows that PsyCap, as a special personal resource, is positively correlated with work engagement [34–36]. Therefore, this study took work engagement as the calibration standard. The scale includes 15 items and three dimensions: vitality, dedication and focus. Likert 7-point scoring method were used with 0 representing “never” and 6 representing “every day” [37]. A higher score indicated a higher level of work engagement. The scale has been widely used to measure the work engagement of nurses, the Cronbach’s α of the scale was greater than 0.9 and has good reliability [37–39]. The higher the correlation coefficient between PsyCap and work engagement, the better the criterion related validity of NPCS.

#### Tests of reliability

Cronbach’s α coefficient, Spearman-Brown split-half reliability, and test–retest reliability were employed. A Cronbach’s α coefficient larger than 0.7 was considered desirable. Test–retest reliability was calculated among the 30 participants who completed the same questionnaire within 2 weeks. Test-retest reliability more than 0.7 was considered desirable [40].

## Results

### Participant characteristics

A total of 619 questionnaires were collected. Most of the participants were women (*n* = 577, 93.2%). The average age for all participants was 32.75 ± 6.35 years and the length of clinical service was 10.16 ± 6.81. 91.1% of nurses held bachelor degree. Table 1 shows the details of nurse participants’ characteristics.

**Table 1:** Demographic characteristics for participants

	Categories	Total sample(*n* = 619, %)	EFA sample(*n* = 319, %)	CFA sample (*n* = 300, %)	Test-retest sample(n = 30, %)
Gender	Male	42(6.8)	24(7.5)	18(6.0)	3(10.0)
	Female	577(93.2)	295(92.5)	282(94.0)	27(90.0)
Age(years)	≤25	85(13.7)	49(15.4)	36(12.0)	17(56.7)
	26 ~ 30	166(26.8)	72(22.6)	94(31.3)	8(26.7)
	31 ~ 35	161(26.0)	82(25.7)	79(26.4)	4(13.3)
	36 ~ 40	149(24.1)	85(26.6)	64(21.3)	0(0)
	≥41	58(18.2)	31(9.7)	27(9.0)	1(3.3)
Clinical career (years)	1 ~ 5	196(31.7)	97(30.4)	99(33.0)	24(80)
	6 ~ 10	164(26.5)	79(24.8)	85(28.3)	5(16.7)
	11 ~ 15	132(21.3)	76(23.8)	56(18.7)	0(0)
	≥16	127(20.5)	67(21.0)	60(20.0)	1(3.3)
Highest education	Secondary technical diploma	1(0.2)	1(0.3)	0(0)	0
	associate degree	39(6.3)	25(7.8)	14(4.7)	0
	bachelor degree	564(91.1)	285(89.3)	279(93.0)	28(93.3)
	Master degree or above	15(2.4)	8(2.5)	7(2.3)	2(6.7)
Marital status	unmarried	147(23.7)	74(23.2)	73(24.3)	21(70.0)
	married	468(75.6)	242(75.9)	226(75.3)	9(30.0)
	Divorce or separation	4(0.6)	3(0.9)	1(0.3)	0(0)
Have Children	No	205(33.1)	106(33.2)	99(33.0)	24(80)
	Yes	414(66.9)	213(66.8)	201(67.0)	6(20)
Employment form	Formal preparation	128(20.7)	68(21.3)	60(20.0)	5(16.7)
	Contract system	476(76.9)	244(76.5)	232(77.3)	19(63.3)
	Personnel agency	15(2.4)	7(2.2)	8(2.7)	6(20.0)
Professional Title	nurse	61(9.9)	35(11.0)	26(8.7)	6(20.0)
	Senior nurse	235(38.0)	108(33.9)	127(42.3)	19(63.3)
	Supervisor nurse	293(47.3)	157(49.2)	136(45.3)	5(16.7)
	vice professor	29(4.7)	18(5.6)	11(3.7)	0(0)
	professor	1(0.2)	1(0.3)	0(0)	0(0)
Work Department	internal medicine	208(33.6)	101(31.7)	107(35.7)	9(30.0)
	Surgery	179(28.9)	95(29.8)	84(28.0)	5(16.7)
	Obstetrics and Gynecology Department	13(2.1)	7(2.2)	6(2.0)	2(6.7)
	pediatrics	19(3.1)	9(2.8)	10(3.3)	3(10.0)
	Emergency Department	26(4.2)	15(4.7)	11(3.7)	1(3.3)
	Operation room	46(7.4)	22(6.9)	24(8.0)	2(6.7)
	ICU	55(8.9)	31(9.7)	24(8.0)	2(6.7)
	Outpatient Department	9(13.0)	3(0.9)	6(2.0)	3(10.0)
	other	64(10.3)	36(11.3)	28(9.3)	3(910.0)
Monthly income (after tax, Yuan)	≤3000	18(2.9)	6(1.9)	12(4.0)	5(16.7)
	3001 ~ 5000	126(20.3)	65(7.0)	61(20.3)	11(36.7)
	5001 ~ 7000	222(35.9)	114(20.4)	108(36.0)	10(33.3)
	7001 ~ 9000	194(31.3)	102(35.7)	92(30.7)	2(6.7)
	≥9001	59(9.5)	32(10.0)	27(9.0)	2(6.7)
Night shift frequency	No	233(37.6)	120(37.6)	113(37.7)	8(26.7)
	Less than four times a month	58(9.4)	32(10.0)	26(8.7)	7(23.3)
	More than four times a month	328(53.0)	167(52.4)	161(53.7)	15(50.0)

One yuan is equal to $0.15

### Item development

According to experts’ opinions, the item “I often encourage myself at work” was deleted. Improved the wording of some items, such as changing item “I will forget unhappy things at work as soon as possible” to “even if I encounter unhappy things at work, I will not care too much”; The item “I can think about problems in my work with critical thinking” was revised to “I can analyze problems in my work with a prudent and rigorous attitude and make judgments and decisions”. Finally, 52 items were retained. The I-CVI of all items was 0.83 to 1.00, greater than 0.78, and the S-CVI/AVE was 0.99, greater than 0.9, indicating that the content validity of the scale is good. During the pilot survey, participants did not report items that were difficult to understand or unclear. All the respondents could complete the questionnaire within 10–15 minutes. Therefore, the items were not further adjusted. Finally, the NPCS with three dimensions and 52 items were developed. There are 27 items of work task-oriented psychological capital, 14 items of interpersonal relationship-oriented psychological capital, and 11 items of learning development-oriented psychological capital.

### Scale development

#### Item analysis

The results of the high-low-27% group method showed that the CR of 52 items ranged from 9.790 to 29.036; the correlations coefficient between the total score of each item and its dimension were 0.575 ~ 0.899, and the corrected item-total correlation coefficients ranged from 0.543 to 0.879; Cronbach’s α coefficients for work task-oriented psychological capital, interpersonal relationship-oriented psychological capital, learning development-oriented psychological capital subscale were 0.973, 0.969 and 0.969 respectively. The Cronbach’s α coefficients after deleting items were 0.971 ~ 0.973, 0.965 ~ 0.968 and 0.965 ~ 0.967 respectively. The commonality of the three subscales was 0.326 ~ 0.816, and the factor load was 0.571 ~ 0.897. All items met the screening criteria, and 52 items were retained.

#### Exploratory factor analysis

A third-round of EFA was conducted to identify the factor structure for NPCS. Results of the first-round EFA showed that the KMO was 0.970 and the Bartlett’s Test of Sphericity was significant (*p* < 0.01).

Combined with the gravel map (Additional file 1), the results showed that there may be 3 ~ 5 factors. The overall structure was ideal when the number of factors was three. The cumulative variance contribution rate was 68.74%, and the correlation coefficients among the three factors were greater than 0.3. It proved that the promax skew method used in this study was reasonable. Combined with the pre conception dimension, factor one was named work task-oriented psychological capital, factor two was named interpersonal relationship-oriented psychological capital, and factor three was named learning development-oriented psychological capital. The loads of 6 items(“Even if I carry out nursing operation independently, I can still consciously adhere to the beliefs and norms of nursing professional ethics”; “I always put patients in the first place, respect and care for patients”; “I try to do my job well, regardless of fame and wealth”; “I can give suggestions and encouragement to the shortcomings of my colleagues’ work”; “I will constantly reflect and summarize the experience and lessons of daily work”; “I can analyze problems in my work and make judgments and decisions with a prudent and rigorous attitude”)on both factors were greater than 0.4, and the load difference was less than 0.25, which were deleted.

In addition, the factor analysis results of four items were different from the original concept dimension. Item 24 “I will actively provide professional nursing services for patients”, item 26 “when there is a conflict between personal interests and work needs, I will choose the latter”, and item 27 “I will stand up in the face of major public health events and disasters” originally belonged to work task-oriented psychological capital. However, results of factor analysis classified it as interpersonal relationship-oriented psychological capital. After discussion by the research group, items 26 and 27 frequently appeared in the previous qualitative interview materials. Combined with semantic analysis, they were still classified as work task-oriented psychological capital, and item 24 was deleted. Item 41 “in collective decision-making, I will take the initiative to put forward my own opinions and suggestions and express my own opinions” originally belonged to interpersonal relationship-oriented psychological capital, and the factor analysis results attributed it to learning development-oriented psychological capital, this item may be related to the “initiative” mentioned in the dimension of learning development-oriented psychological capital. According to the discussion of the research group, the connotation of initiative as “actively learn knowledge and study hard according to their own career development plan”, which is inconsistent with the connotation of initiative as, so it is still classified as interpersonal relationship-oriented psychological capital.

Seven items were deleted in the first EFA. The remaining 45 items were analyzed further, and 3 factors were extracted, explaining 68.65% of the variance (KMO = 0.967, Bartlett’s Test of Sphericity was significant). In the second EFA, the loads of the two items (“I feel happy and proud to be a member of the hospital or department team”; “I am good at observing and thinking about details in my work”) on both factors were greater than 0.4, and the load difference was less than 0.25, which were deleted.

Two items were deleted in the second EFA. The remaining 43 items were furtherly analyzed, and 3 factors were extracted, explaining 68.71% of the variance (KMO = 0.967, Bartlett’s Test of Sphericity was significant). In the third EFA, the load of each item under its own factor was greater than 0.40, which showed a good structural validity (Table 2).

**Table 2:** Exploratory factor analysis of the NPCS

Item	F1	F1	F3	Commonality
8	0.873			0.675
11	0.833			0.715
9	0.830			0.729
6	0.796			0.635
21	0.792			0.623
10	0.779			0.671
12	0.779			0.666
7	0.773			0.633
16	0.766			0.721
14	0.756			0.750
15	0.742			0.507
18	0.738			0.680
13	0.734			0.556
4	0.733			0.581
3	0.707			0.505
19	0.697			0.649
17	0.666			0.698
5	0.629			0.615
20	0.595			0.662
2	0.576			0.437
1	0.460			0.338
33		1.029		0.839
34		1.018		0.826
32		0.912		0.831
38		0.903		0.774
35		0.859		0.745
39		0.827		0.723
30		0.784		0.756
31		0.737		0.772
36		0.717		0.718
28		0.700		0.690
27		0.604		0.564
29		0.575		0.707
26		0.572		0.602
51			0.976	0.838
50			0.973	0.821
48			0.775	0.823
52			0.767	0.745
49			0.729	0.799
47			0.605	0.811
46			0.604	0.769
45			0.536	0.743
41			0.464	0.604

Through item analysis and EFA, 43 items were retained, including 23 items for work task-oriented psychological capital dimension, 11 items for interpersonal relationship-oriented psychological capital and 9 items for learning development-oriented psychological capital.

### Scale validation

#### Confirmatory factor analysis

CFA was performed to validate the factor structure obtained from EFA. Firstly, the collected data were tested for normality test. As shown in Table 3, the skewness index of each item was far less than 3, and the kurtosis index was far less than 8, which conformed to the normal distribution [41]. Therefore, the maximum likelihood method was used for CFA.

**Table 3:** Normality test of confirmatory factor analysis

Item	Skewness coefficient	Kurtosis coefficient	Item	Skewness coefficient	Kurtosis coefficient
1	−0.789	1.285	27	−0.679	−0.368
2	−0.632	0.924	28	−0.330	− 0.815
3	− 0.355	0.126	29	−0.208	−0.826
4	−0.568	0.993	30	−0.368	− 0.486
5	−0.753	0.789	31	− 0.419	−0.807
6	−0.567	0.072	32	−0.420	− 0.386
7	− 0.805	0.479	33	−0.847	0.324
8	−0.414	−0.002	34	−0.586	− 0.459
9	−0.824	1.145	35	−0.380	−0.593
10	−0.501	0.279	36	− 0.513	−.405
11	−0.282	− 0.464	38	−0.619	− 0.392
12	− 0.451	0.025	39	−0.743	0.779
13	−0.318	− 0.051	41	−0.319	− 0.595
14	−0.503	0.285	45	−0.298	− 0.535
15	−0.450	0.076	46	−0.412	− 0.426
16	−0.239	0.230	47	−0.343	−0.646
17	−0.285	−0.344	48	−0.225	− 0.680
18	−0.023	− 0.042	49	− 0.241	− 0.588
19	− 0.138	0.076	50	−0.089	− 0.305
20	−0.217	− 0.544	51	0.054	− 0.661
21	−0.305	− 0.179	52	− 0.225	− 0.491
26	−0.592	− 0.006			

As shown in Table 4, the error variance of the estimated values of each parameter was positive, the t-test was statistically significant (*p* < 0.001), the standard error was 0.050 ~ 0.163, the factor load was 0.524 ~ 0.910, and the basic fitness was good. Overall model fitting index *x*^2^/*df* =3.524, RMR = 0.044, RMSEA = 0.092, IFI = 0.822, TLI = 0.812, CFI = 0.822, PNFI = 0.729, the RMSEA was greater than 0.08, which was not ideal. According to the modified index (MI): e1-e2 was 118.851, e6-e8 was 110.047, e50-e51 was 82.877, e18–e19 was 81.773, e46-e47 was 77.260, and e33-e34 was 66.673, six covariance correlations were gradually added. The results showed that each fitting index *x*^2^/*df* =2.839, RMR =0.041, RMSEA =0.078, IFI =0.872, TLI =0.863, CFI =0.871, and PNFI =0.768, which were within the acceptable range. The results indicated that the overall fit between the model and the scale was good. The final modified model was shown in Additional file 2.

**Table 4:** Parameter estimation of confirmatory factor analysis

	Regression Weights Estimate	Standard error	*P-*value	Factored load	CR	AVE
v1 ← work task-oriented	1.000			0.571	0.953	0.471
v2 ← work task-oriented	1.084	0.119	9.094^***^	0.611		
v3 ← work task-oriented	1.246	0.127	9.842^***^	0.705		
v4 ← work task-oriented	1.206	0.122	9.909^***^	0.699		
v5 ← work task-oriented	1.242	0.124	9.982^***^	0.719		
v6 ← work task-oriented	1.495	0.163	9.154^***^	0.631		
v7 ← work task-oriented	1.540	0.154	10.018^***^	0.731		
v8 ← work task-oriented	1.525	0.162	9.385^***^	0.658		
v9 ← work task-oriented	1.570	0.144	10.887^***^	0.833		
v10 ← work task-oriented	1.598	0.149	10.761^***^	0.817		
v11 ← work task-oriented	1.710	0.163	10.491^***^	0.792		
v12 ← work task-oriented	1.538	0.153	10.023^***^	0.736		
v13 ← work task-oriented	1.383	0.139	9.937^***^	0.722		
v14 ← work task-oriented	1.410	0.132	10.651^***^	0.807		
v15 ← work task-oriented	1.350	0.154	8.788^***^	0.612		
v16 ← work task-oriented	1.183	0.127	9.329^***^	0.660		
v17 ← work task-oriented	1.150	0.119	9.666^***^	0.690		
v18 ← work task-oriented	0.994	0.125	7.959^***^	0.524		
v19 ← work task-oriented	1.162	0.137	8.499^***^	0.575		
v20 ← work task-oriented	1.144	0.121	9.437^***^	0.672		
v21 ← work task-oriented	1.242	0.135	9.219^***^	0.651		
v26 ← work task-oriented	1.107	0.129	8.576^***^	0.593		
v27 ← work task-oriented	1.149	0.122	9.407^***^	0.670		
v28 ← interpersonal relationship-oriented	1.000			0.824	0.968	0.712
v29 ← interpersonal relationship-oriented	0.948	0.059	16.132^***^	0.790		
v30 ← interpersonal relationship-oriented	0.983	0.052	18.824^***^	0.870		
v31 ← interpersonal relationship-oriented	1.049	0.057	18.338^***^	0.853		
v32 ← interpersonal relationship-oriented	0.996	0.050	19.807^***^	0.891		
v33 ← interpersonal relationship-oriented	1.030	0.053	19.431^***^	0.871		
v34 ← interpersonal relationship-oriented	1.015	0.053	19.081^***^	0.860		
v35 ← interpersonal relationship-oriented	1.015	0.054	18.858^***^	0.871		
v36 ← interpersonal relationship-oriented	1.059	0.054	19.490^***^	0.889		
v38 ← interpersonal relationship-oriented	1.045	0.056	18.652^***^	0.869		
v39 ← interpersonal relationship-oriented	0.957	0.058	16.644^***^	0.804		
v41 ← interpersonal relationship-oriented	0.927	0.064	14.509^***^	0.735		
v45 ← learning development-oriented	1.000			0.753	0.958	0.758
v46 ← learning development-oriented	1.229	0.073	16.832^***^	0.857		
v47 ← learning development-oriented	1.322	0.074	17.747^***^	0.909		
v48 ← learning development-oriented	1.349	0.079	17.026^***^	0.910		
v49 ← learning development-oriented	1.274	0.080	15.958^***^	0.871		
v50 ← learning development-oriented	1.224	0.080	15.302^***^	0.824		
v51 ← learning development-oriented	1.161	0.075	15.569^***^	0.832		
v52 ← learning development-oriented	1.179	0.075	15.823^***^	0.852		

^***^ *p*<0.001

### Convergent and discriminant validity

The three-dimensional CR for work task-oriented, interpersonal relationship-oriented and learning development-oriented psychological capital were 0.953, 0.968 and 0.958, respectively. The AVE were 0.471, 0.712 and 0.758, which indicated that the internal quality of the model was good and the structural dimension of the NPCS had good convergent validity (Table 4).

Table 5 shows that the square root of AVE under the dimension of work task-oriented psychological capital was 0.686, and the correlation coefficients between this dimension and interpersonal relationship-oriented psychological capital and learning development-oriented psychological capital were 0.760 and 0.733 respectively; the square root of AVE under the dimension of interpersonal relationship-oriented psychological capital was 0.844, and the correlation coefficients between this dimension and learning development-oriented psychological capital and work task-oriented psychological capital were 0.757 and 0.760 respectively; the square root of AVE under the dimension of learning development-oriented psychological capital was 0.871, and the correlation coefficients between this dimension and work task-oriented psychological capital and interpersonal relationship-oriented psychological capital were 0.733 and 0.757 respectively. Except that the correlation coefficient between the work task-oriented psychological capital dimension and the other two dimensions was greater than the square root of AVE under this dimension, and the discriminant validity was poor, the correlation coefficient of other dimensions was less than the square root of AVE, indicating that the discriminant validity of the scale was acceptable.

**Table 5:** Discriminant validity of the NPCS

	work task-oriented	interpersonal relationship-oriented	learning development-oriented
Work task-oriented	0.471(AVE)		
Interpersonal relationship-oriented	0.760	0.712(AVE)	
Learning development-oriented	0.733	0.757	0.758(AVE)
Square root of AVE	0.686	0.844	0.871

### Criterion-related validity

The correlation coefficients between the factor scores, total scores of the NPCS and the total score of the work engagement scale were 0.605, 0.443, 0.453 and 0.579 respectively (each *p* < 0.01).

### Tests of reliability

The Cronbach’s *α* of the overall NPCS was 0.975 and that of each sub-factor ranged from 0.952 to 0.967; The Spearman-Brown split-half reliability for the overall NPCS was 0.872 and that for each sub-factor ranged from 0.897 to 0.945; The test-retest reliability of the overall NPCS was 0.850 and that of each sub-factor ranged from 0.711 to 0.851 (Table 6).

**Table 6:** Reliability of the NPCS

	Cronbach’s α	Spearman-Brown split-half reliability	test-retest reliability
Work task-oriented psychological capital	0.952	0.897	0.851
interpersonal relationship-oriented psychological capital	0.967	0.945	0.711
learning development-oriented psychological capital	0.955	0.925	0.779
Total	0.975	0.872	0.850

## Discussion

This is the first study to explore the structure of PsyCap in Chinese nursing group and develop a self-report NPCS measurement to evaluate nurses’ PsyCap. Three dimensions for NPCS have been summarized as follows: work task-oriented psychological capital, interpersonal relationship-oriented psychological capital, learning development-oriented psychological capital. The results of this study indicates that the NPCS is a valid and reliable scale for assessing PsyCap among Chinese nurses.

In this study, the 43-item NPCS was developed through item development, scale development, and scale validation. In the course of item development, an item pool with 52 items was developed through literature analysis, qualitative interviews, Delphi expert correspondence and pilot survey.

During scale development, according to the exclusion criteria of item analysis and EFA, 9 items were deleted through group discussion. The results of EFA showed that the NPCS included three dimensions of “work task-oriented psychological capital, interpersonal relationship-oriented psychological capital and learning development-oriented psychological capital”, explaining 68.71% of the total variance. However, such secondary dimensions as “calmness” and “work immersion” had not been explored. This might be related to sample size and sample distribution. The sample size of EFA in this study was only convenient sampling 319 nurses from two tertiary hospitals in HeBei, China. The representativeness of the sample was limited to a certain extent, which might lead to certain bias in the survey results.

During scale validation, the initial model fit of CFA was not ideal (RMSEA = 0.092>0.08). Six covariance correlations were added according to the modified index. Each covariance correlation was between the residuals of different items in the same dimension. There was no cross dimension. It conforms to the preset model and the correlation can be explained. After correction, the fitting indexes (*x*^2^/*df* =2.839, RMR = 0.041, RMSEA = 0.078, IFI = 0.872, TLI = 0.863, CFI = 0.871, PNFI = 0.768) were all within the acceptable range. Of the CFA results for confirming construct validity, the discrimination validity of work task-oriented psychological capital dimension was poor, and its square root of AVE was smaller than the correlation between factors. The possible reason was that the positive psychological power held by the nurses in the process of interpersonal relationship processing and learning and development can further promote the completion of work tasks, so that the NPCS had insufficient discrimination in the dimension of work task-oriented psychological capital [42]. But in general, the three factor structure of NPCS was acceptable. The I-CVI of 43 items and the S-CVI/AVE in the final scale were all good, which indicated that the content validity of the scale is good.

The three dimensions of NPCS differs from other scales because it indicates not only the PsyCap characteristics of nurses when they complete their work tasks, but also the positive psychological strength of nurses in the process ofinterpersonal communication and learning development. First, the work task-oriented psychological capital reflects the positive psychological power of nurses tomotivate and adjust individuals in the process of successfully completing nursing tasks or coping with task-related setbacks [23]. To a certain extent, it is in line with the content of the existing nurses’ PsyCap questionnaire and reflects the cross-cultural effectiveness. “When personal interests conflict with work needs, I will choose the latter” and “In the face of major public health events and disasters, I will step forward” reflect the dedication of nurses. This spirit of dedication is highly consistent with the Taoist concept of humanistic care, which fully reflects the characteristics of Chinese collectivist culture. However, in the complex medical care environment, in addition to showing a positive psychological state when completing specific work, it is also very necessary to have good interpersonal skills. Second, the relationship-oriented psychological capital is an innovative concept in the study of PsyCap localization, which reflects the positive psychological strength required by nurses to coordinate the doctor-nurse-patient relationship, communicate related events, and interact effectively with the organization [21]. Nurses’ interpersonal relationship in work has the nature of ordinary employees’ interpersonal relationship, but also has its characteristics. Dealing with the relationships with patients, doctors and nurses is conducive to improving work efficiency and promoting the smooth development of work [43, 44]. “I treat people with tolerance in my work and life, and I don’t square accounts in every detail”, “When colleagues’ views and opinions contradict my own, I can listen carefully and draw useful suggestions” reflect the unity and inclusiveness in the Confucian classics. At the same time, some items also reflect the local ideas of modesty and teamwork in China’s traditional culture, such as “When encountering problems that I don’t understand, I will humbly ask others for advice”, “My colleagues and me can help each other and work together”. In addition, nursing profession takes innovation as the soul, self- transcendence and lifelong learning as the goal, and integrates self-learning with organizational learning, so as to continuously improve personal quality and promote the competitiveness of individuals and hospitals in the medical market [45]. The dimension of learning development-oriented psychological capital in this scale reflects the positive psychological ability of nurses in the process of professional learning, individual planning and self-goal realization. This dimension is similar to the results of Hou et al. [46].

However, there are some limitations to this study. First, the nurse participants recruited in the study were only from two tertiary hospitals in HeBei, China. The representativeness of the participants was limited. It is suggested that future research conduct multi-center survey with a larger sample to establish a national norm and verify the applicability of the NPCS in nurses. Second, this study adopted the convenient sampling method, which may affect the universality of the study. Therefore, future studies are recommended a random stratification method to test the universality of NPCS. Finally, this study only includes work engagement as the calibration standard, and its measured ability may not fully reflect the three core constructs of the NPCS. Future studies should consider to select Calibration association validity such as “traditionality”, “interdependent”, “contextual performance”, “learning performance”, “innovation performance”, so as to further test the effectiveness of the NPCS.

## Conclusions

To our knowledge, the NPCS is the first scale that comprehensively considers the Chinese cultural background and nursing professional characteristics to assess nurses’ PsyCap. The NPCS has good reliability and validity. It is suggested that clinical nurses and nursing managers use the NPCS to measure the levels of PsyCap in nurses. Moreover, the assessment results can be quantified as a guide for improving the ability of PsyCap, which is conducive to improving individual competitiveness and organizational performance.

## Supplementary Information

**Additional file 1.** Scree Plot based on EFA of the NPCS.

**Additional file 2.** Confirmatory factor analysis of the NPCS. Note: F1 = work task-oriented psychological capital; F2 = interpersonal relationship-oriented psychological capital; F3 = learning development-oriented psychological capital.

**Additional file 3.** The Nurse Psychological Capital Scale (NPCS).

## Data Availability

The dataset in this study is available from the corresponding author on reasonable request.
