# Supplementary figures and images for: Development and initial validation of the psychological capital scale for nurses in Chinese local context
Source: BMC Nurs. 2023 Feb 2;22:28. doi: 10.1186/s12912-022-01148-x (PMC9893552; doi:10.1186/s12912-022-01148-x)

&[ ]

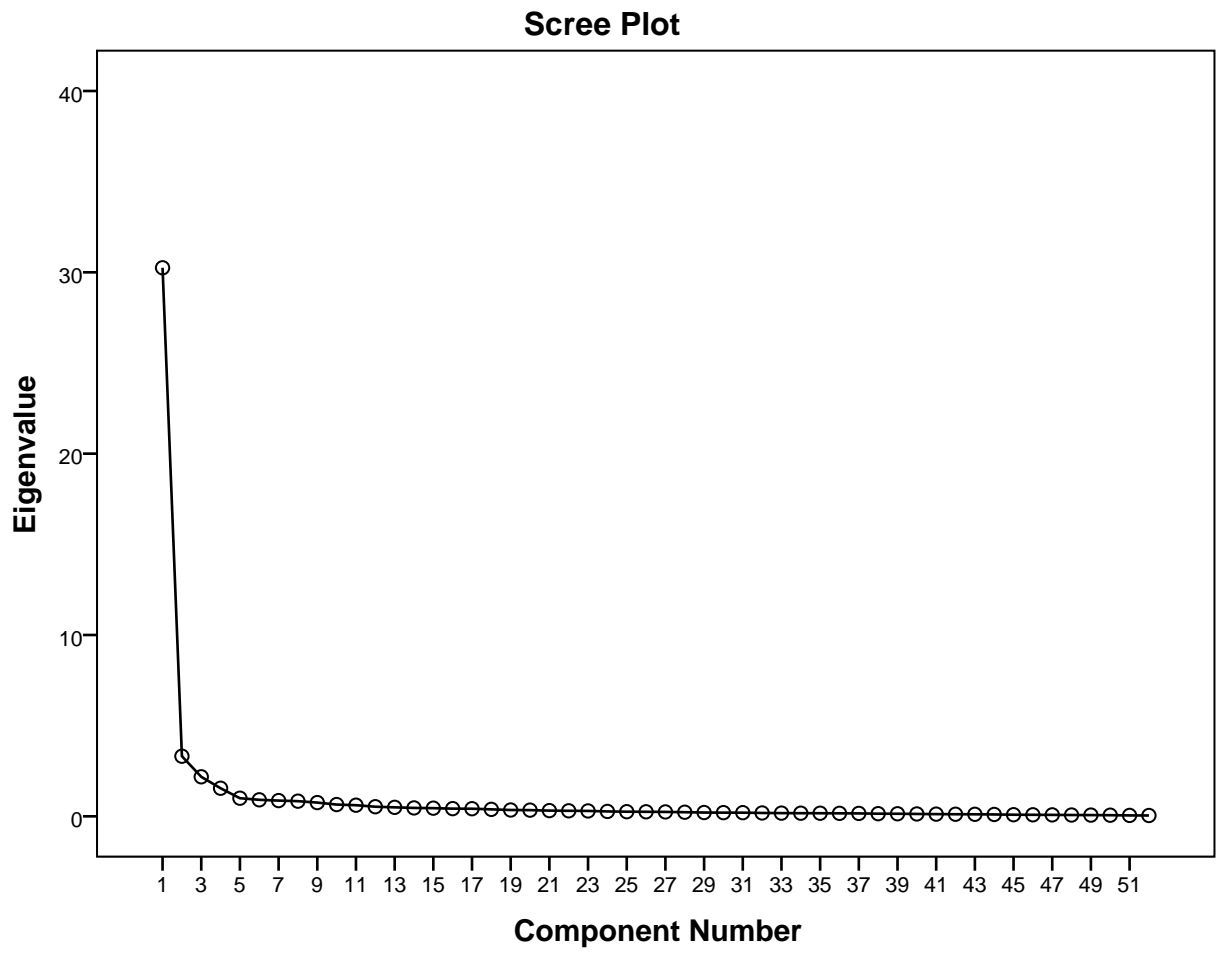

Supplement: Supplementary file 1 — Additional file 1. Scree Plot based on EFA of the NPCS. [file 12912_2022_1148_MOESM1_ESM.pdf]
