# Supplementary material for: Development and initial validation of the psychological capital scale for nurses in Chinese local context
Source: BMC Nurs. 2023 Feb 2;22:28. doi: 10.1186/s12912-022-01148-x (PMC9893552; doi:10.1186/s12912-022-01148-x)
Supplement: Supplementary file 2 — Additional file 2. Confirmatory factor analysis of the NPCS. Note: F1 = work task-oriented psychological capital; F2 = interpersonal relationship-oriented psychological capital; F3 = learning development-oriented psychological capital. [file 12912_2022_1148_MOESM2_ESM.pdf]

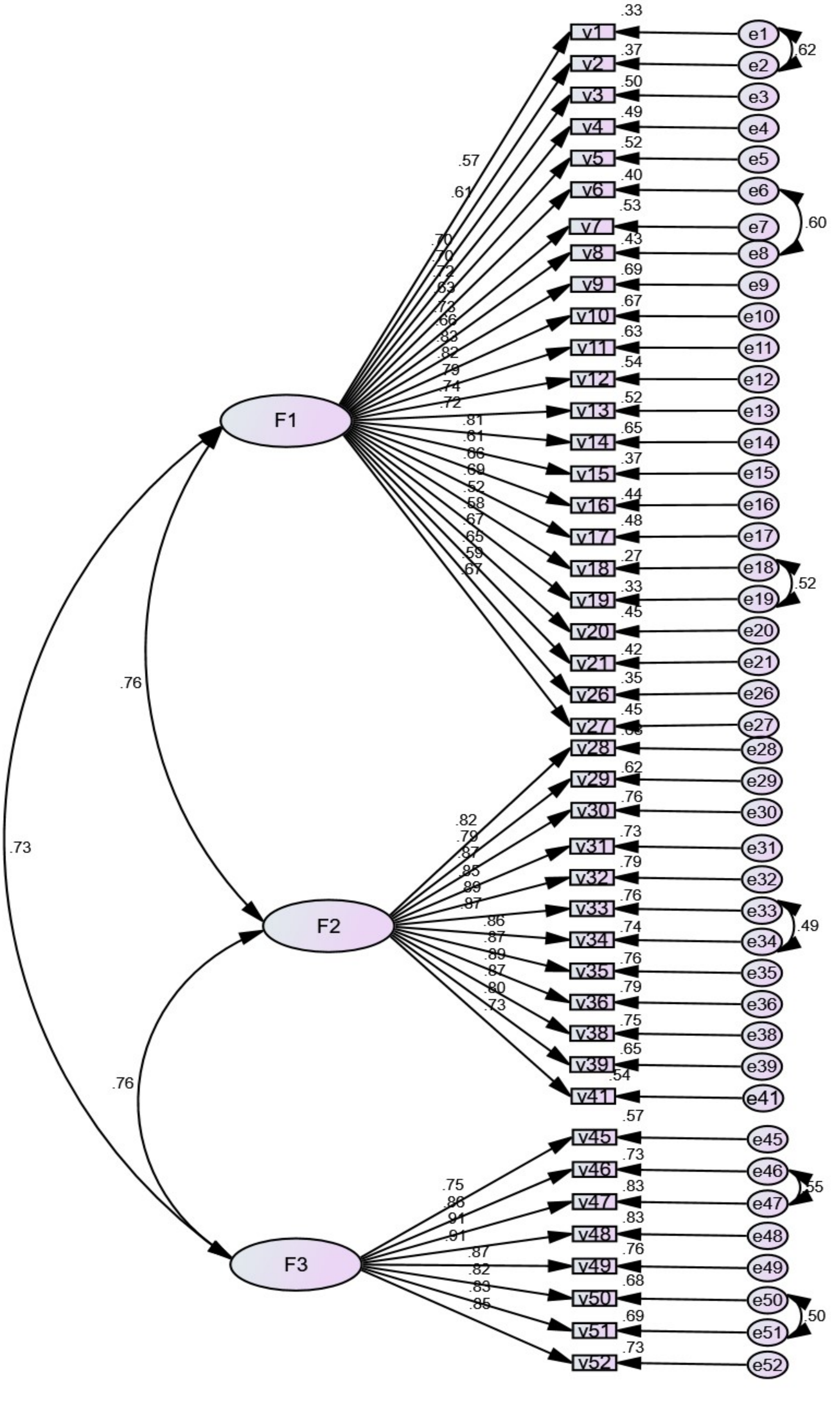

Fig. 2 Confirmatory factor analysis of the NPCS

Note: F1=work task-oriented psychological capital; F2=interpersonal relationship-oriented psychological capital; F3=learning development-oriented psychological capital
