# Supplementary material for: Development and initial validation of the psychological capital scale for nurses in Chinese local context
Source: BMC Nurs. 2023 Feb 2;22:28. doi: 10.1186/s12912-022-01148-x (PMC9893552; doi:10.1186/s12912-022-01148-x)
Supplement: Supplementary file 3 — Additional file 3. The Nurse Psychological Capital Scale (NPCS). [file 12912_2022_1148_MOESM3_ESM.docx]

Additional file 3. The NPCS

|  | Strongly disagree | Disagree | Neutral | Agree | Strongly agree |
| --- | --- | --- | --- | --- | --- |
| **work task-oriented psychological capital** | | | | | |
| 1. I can maintain a strong sense of risk and prevent accidents. | 1 | 2 | 3 | 4 | 5 |
| 2. In the face of emergencies, I can calmly and proactively respond | 1 | 2 | 3 | 4 | 5 |
| 3. I can control my emotions well in the work situati-on. | 1 | 2 | 3 | 4 | 5 |
| 4. In the face of challenging tasks, I will consider and prepare from various aspects in combination with my own abilities. | 1 | 2 | 3 | 4 | 5 |
| 5. I can keep my whole heart and soul in every  nursing work. | 1 | 2 | 3 | 4 | 5 |
| 6. As a nurse, I am sincerely proud of this great  cause. | 1 | 2 | 3 | 4 | 5 |
| 7. After finishing my work, I often feel a sense of accomplishment. | 1 | 2 | 3 | 4 | 5 |
| 8. I enjoy working very much. | 1 | 2 | 3 | 4 | 5 |
| 9. I will actively and bravely face the problems and difficulties encountered in my work. | 1 | 2 | 3 | 4 | 5 |
| 10. I am a person who can bear hardships. The more I get into trouble, the more tenacious I become. | 1 | 2 | 3 | 4 | 4 |
| 11. No matter how difficult and tired the work is, I  will stick to it. | 1 | 2 | 3 | 4 | 5 |
| 12. For things with uncertain outcome, I prefer to  think in a positive way. | 1 | 2 | 3 | 4 | 5 |
| 13. I believe that I am an important factor in successfully completing various tasks. | 1 | 2 | 3 | 4 | 5 |
| 14. I can treat work with a positive attitude. | 1 | 2 | 3 | 4 | 5 |
| 15. Even if I encounter unhappy things in my work, I will not pay too much attention to them. | 1 | 2 | 3 | 4 | 5 |
| 16. For unhappy things, I can always analyze them in many ways and see the positive side. | 1 | 2 | 3 | 4 | 5 |
| 17. I have confidence in my ability to work. | 1 | 2 | 3 | 4 | 5 |
| 18. I like to set higher goals for myself. | 1 | 2 | 3 | 4 | 5 |
| 19. I like to do challenging work. | 1 | 2 | 3 | 4 | 5 |
| 20. I can constantly pursue the perfection of my work and strive to do my best. | 1 | 2 | 3 | 4 | 5 |
| 21. I can keep a good balance between work and life. | 1 | 2 | 3 | 4 | 5 |
| 22. When personal interests conflict with work needs, I will choose the latter. | 1 | 2 | 3 | 4 | 5 |
| 23. In the face of major public health events and  disasters, I will step forward. | 1 | 2 | 3 | 4 | 5 |
| **interpersonal relationship-oriented psychological capital** | | | | | |
| 24. I treat people with tolerance in my work and life, and I don't square accounts in every detail. | 1 | 2 | 3 | 4 | 5 |
| 25. When patients or family members misunderstand  my work, I can think in terms of others and respond to their questions in a timely manner. | 1 | 2 | 3 | 4 | 5 |
| 26. When colleagues' views and opinions contradict my own, I can listen carefully and draw useful  suggestions. | 1 | 2 | 3 | 4 | 5 |
| 27. I will patiently listen to and answer all kinds of  questions raised by patients and their families. | 1 | 2 | 3 | 4 | 5 |
| 28. I can face up to my shortcomings and deficiencies in my work. | 1 | 2 | 3 | 4 | 5 |
| 29. When encountering problems that I don't  understand, I will humbly ask others for advice. | 1 | 2 | 3 | 4 | 5 |
| 30. I am willing to constantly learn from others' strengths to make up for my own shortcomings. | 1 | 2 | 3 | 4 | 5 |
| 31. I am good at discovering the advantages of others. | 1 | 2 | 3 | 4 | 5 |
| 32. Face the criticism and accusation of others, correct mistakes if you have made any and guard against them if you have not. | 1 | 2 | 3 | 4 | 5 |
| 33. My colleagues and me can help each other and work together. | 1 | 2 | 3 | 4 | 5 |
| 34. I often affirm my colleagues' achievements and performance. | 1 | 2 | 3 | 4 | 5 |
| 35. In collective decision-making, I will actively put  forward my own opinions and suggestions. | 1 | 2 | 3 | 4 | 5 |
| **learning development-oriented psychological capital** | | | | | |
| 36. I can question inappropriate nursing measures according to my professional knowledge, further reason and think, and make the best nursing decision for patients. | 1 | 2 | 3 | 4 | 5 |
| 37. I can actively learn new nursing knowledge and  operating skills. | 1 | 2 | 3 | 4 | 5 |
| 38. I can actively learn other fields related to nursing. | 1 | 2 | 3 | 4 | 5 |
| 39.In terms of work and study, I can take the initiative to study hard, rather than being satisfied with completing tasks. | 1 | 2 | 3 | 4 | 5 |
| 40.I can actively formulate career development plans  according to my own situation and make efforts for  them. | 1 | 2 | 3 | 4 | 5 |
| 41. I can use leading-edge nursing knowledge and operating skills to propose new nursing plans. | 1 | 2 | 3 | 4 | 5 |
| 42. When I encounter new problems in my work, I  can break the traditional thinking and take the initiative to find new scientific methods to solve problems. | 1 | 2 | 3 | 4 | 5 |
| 43. I am willing to try new methods and ideas in my work. | 1 | 2 | 3 | 4 | 5 |
